# Time-Resolved Investigation of Molecular Components Involved in the Induction of ${\mathrm{NO}}_{3}^{–}$ High Affinity Transport System in Maize Roots

**DOI:** 10.3389/fpls.2016.01657

**Published:** 2016-11-08

**Authors:** Youry Pii, Massimiliano Alessandrini, Luca Dall’Osto, Katia Guardini, Bhakti Prinsi, Luca Espen, Anita Zamboni, Zeno Varanini

**Affiliations:** ^1^Faculty of Science and Technology, Free University of BolzanoBolzano, Italy; ^2^Department of Biotechnology, University of VeronaVerona, Italy; ^3^Department of Agricultural and Environmental Sciences - Production, Landscape, Agroenergy, University of MilanoMilano, Italy

**Keywords:** maize, NO^-^_3_ induction, ZmNRT2.1, ZmNRT3.1A, PM H^+^-ATPase, protein complexes

## Abstract

The induction, i.e., the rapid increase of nitrate (${\mathrm{NO}}_{3}^{–}$) uptake following the exposure of roots to the anion, was studied integrating physiological and molecular levels in maize roots. Responses to ${\mathrm{NO}}_{3}^{–}$ treatment were characterized in terms of changes in ${\mathrm{NO}}_{3}^{–}$ uptake rate and plasma membrane (PM) H^+^-ATPase activity and related to transcriptional and protein profiles of *NRT2, NRT3*, and *PM H^+^-ATPase* gene families. The behavior of transcripts and proteins of *ZmNRT2s* and *ZmNRT3s* suggested that the regulation of the activity of inducible high-affinity transport system (iHATS) is mainly based on the transcriptional/translational modulation of the accessory protein ZmNRT3.1A. Furthermore, ZmNRT2.1 and ZmNRT3.1A appear to be associated in a ∼150 kDa oligomer. The expression trend during the induction of the 11 identified *PM H^+^-ATPase* transcripts indicates that those mainly involved in the response to ${\mathrm{NO}}_{3}^{–}$ treatment are *ZmHA2* and *ZmHA4*. Yet, partial correlation between the gene expression, protein levels and enzyme activity suggests an involvement of post-transcriptional and post-translational mechanisms of regulation. A non-denaturing Deriphat-PAGE approach allowed demonstrating for the first time that PM H^+^-ATPase can occur *in vivo* as hexameric complex together with the already described monomeric and dimeric forms.

## Introduction

Nitrate (${\mathrm{NO}}_{3}^{–}$) is the major nitrogen (N) source used by plants growing in well-aerated agricultural soils and plants adsorb it with a complex multi-component uptake machinery. Nitrate uptake kinetic, measured as a function of the external ${\mathrm{NO}}_{3}^{–}$ concentration, shows a biphasic behavior due to the existence of at least two different transport mechanisms (Forde and Clarkson, 1999). At low ${\mathrm{NO}}_{3}^{–}$ concentration (less than 1 mM), plants absorb the anion thanks to a high affinity transport system (HATS), which displays a saturable kinetics that can be described by a Michaelis Menten kinetic (Filleur et al., 2001). On the other hand, when the external concentration of ${\mathrm{NO}}_{3}^{–}$ is higher than 0.5-1 mM, it is taken up with a non-saturable kinetic *via* the low affinity transport system (LATS) (Touraine and Glass, 1997).

In plants, proteins involved in the ${\mathrm{NO}}_{3}^{–}$ uptake at root plasma membrane (PM) level have been identified and they are mostly encoded by two gene families, namely *NRT1* and *NRT2* (Nacry et al., 2013). Whilst *NRT1* genes encode members of LATS, *NRT2* genes encode high affinity transporters (Plett et al., 2010), which have already been characterized in both herbaceous and woody plant species (Ranamalie Amarasinghe et al., 1998; Huang et al., 1999; Fraisier et al., 2000; Vidmar et al., 2000; Cai et al., 2008; Feng et al., 2011; Pii et al., 2014). However, it has also been observed that, among the high affinity transporters, the major role is played by NRT2.1, whereas NRT2.2 and NRT2.4 give a minor contribution to the ${\mathrm{NO}}_{3}^{–}$ uptake in the high affinity concentration range (Filleur et al., 2001; Li et al., 2007; Kiba et al., 2012). In addition, several pieces of evidence highlighted that the components of HATS are often co-expressed with the accessory protein NRT3, which is required for a functioning ${\mathrm{NO}}_{3}^{–}$ transport (Zhou et al., 2000; Tong et al., 2005; Okamoto et al., 2006).

In agricultural soils, the concentration of ${\mathrm{NO}}_{3}^{–}$ fluctuates as a function of time and space, therefore plants have adapted their uptake system so that it could be modulated by the bioavailability of ${\mathrm{NO}}_{3}^{–}$. This system, known as induction, was first described by Jackson et al. (1973) and it was shown to involve the activity of inducible HATSs (Siddiqi et al., 1989). The exposure to ${\mathrm{NO}}_{3}^{–}$ causes in plant roots an expression burst of those genes encoding members of HATS, in particular *NRT2.1* and *NRT2.2* (Zhuo et al., 1999; Okamoto et al., 2003) and results in a higher rate of anion uptake. After the induction, the ${\mathrm{NO}}_{3}^{–}$ uptake rate reaches a peak, within hours in herbaceous species and days in tree plants (Kronzucker et al., 1995; Min et al., 1998; Pii et al., 2014), then rapidly declines, due to negative feedback mechanisms (Glass et al., 2001). Furthermore, it has been also observed that different maize inbred lines (T250 and Lo5) are characterized by different induction time, despite being exposed to the same concentration of nitrate (Zamboni et al., 2014).

The uptake of ${\mathrm{NO}}_{3}^{–}$ is an active transport system requiring the input of metabolic energy (Siddiqi et al., 1990; Glass et al., 1992). Electrophysiological studies highlighted that the ${\mathrm{NO}}_{3}^{–}$ uptake occurs as a symport together with H^+^, in which the metabolic energy is needed to maintain the proton gradient through the activity of PM H^+^-ATPase (McClure et al., 1990a,b; Glass et al., 1992; Santi et al., 1995; Espen et al., 2004). The PM H^+^-ATPases are members of the P-type ATPases superfamily, which are characterized by their ability to pump ions across the cellular membranes. In turn, the P-type superfamily is subdivided into five subfamilies (P1 to P5), which encompasses enzymes with different substrate specificities (Axelsen and Palmgren, 1998; Palmgren and Nissen, 2011). The PM H^+^-ATPase enzymes involved in the mineral nutrition of plants belong to the P3 subfamily, which has been so far characterized only in plant and fungi (Pedersen et al., 2012).

Data obtained in at least a decade show that, in the specific case of ${\mathrm{NO}}_{3}^{–}$ uptake, the time course of the PM H^+^-ATPase activity follows the same profile of that displayed by the ${\mathrm{NO}}_{3}^{–}$ uptake, as well as the negative feed-back regulation following the maximum rate of nitrate influx (Santi et al., 2003; Nikolic et al., 2012). Furthermore, it was also hypothesized that PM H^+^-ATPases might play a role in the uptake of ${\mathrm{NO}}_{3}^{–}$, suggesting that plants might satisfy their requirements by modulating the expression of different genes in response to the fluctuations of ${\mathrm{NO}}_{3}^{–}$ concentration in the rhizosphere (Santi et al., 2003; Sorgonà et al., 2011; Nikolic et al., 2012). However, several lines of evidence showed that the oligomerization is a common feature shared by the members of the P-type ATPase family of pumps, suggesting that, beside a transcriptional control of genes, a post-translational control of the activity might also be possible. Early studies in *Neurospora crassa* assessed that the functional unit of the PM H^+^-ATPase reconstituted in liposomes with an excess of lipids might be a monomer (Goormaghtigh et al., 1986). On the other hand, other authors demonstrated the dimeric and hexameric occurrence of PM H^+^-ATPases, even though their functional roles remain to be clarified (Briskin and Reynolds-Niesman, 1989; Kanczewska et al., 2005; Ottmann et al., 2007). As mentioned above, the P-type PM H^+^-ATPases have a regulatory domain located at the C-terminus, known as R domain (Pedersen et al., 2007), through which the activation of the pumps is modulated by a phosphorylation-dependent binding of the 14-3-3 regulatory protein to this domain (Jelich-Ottmann et al., 2001; Fuglsang et al., 2003). The three-dimensional reconstruction of the purified PM H^+^-ATPase/14-3-3 complex highlighted a hexameric aggregation (Ottmann et al., 2007), further confirming previous findings obtained through crystallography-based techniques (Huang and Berry, 1990; Auer et al., 1998). Nevertheless, information concerning the correlation between the multimeric arrangement of PM H^+^-ATPases and their activation is still lacking. In a recent work, Justesen et al. (2013) proposed that the active form of the *Arabidopsis thaliana* PM H^+^-ATPase 2 (AHA2) might be the monomeric one. However, in this specific case the authors used for their investigations a recombinant protein devoid of the R domain, thus constitutively active, which might be rather far from the physiological conditions.

On the bases of these premises, the aim of the present work was the characterization of the iHATS in maize roots by coupling the changes in ${\mathrm{NO}}_{3}^{–}$ uptake rate and ATP hydrolysing activity, with changes in protein and transcriptional levels during the anion treatment (0-24 h), in order to gain a comprehensive view at different levels. In addition, the separation of protein complexes in a non-denaturing Deriphat-PAGE and protein identification by mass spectrometry allowed us shedding light in the supramolecular organization of the molecular entities involved in the high affinity ${\mathrm{NO}}_{3}^{–}$ transport at PM.

## Materials and Methods

### Plant Material and Growth Conditions

Seeds of *Zea mais* L. (hybrid PR33T56, Pioneer Hi-Bred Italia S.r.l) previously soaked in running water for 24 h, were allowed to germinate in the dark at 26°C for 72 h over an aerated solution of 0.5 mM CaSO_4_. Seedlings were transferred to plastic pots containing 2.2 L of 0.5 mM CaSO_4_ solution (12 seedlings in each pot) and were grown for 24 h in the dark at 80% relative humidity and 26°C. Seedlings were then grown using a nutrient solution (NS) containing 100 μM MgSO_4_, 200 μM K_2_SO_4_, 400 μM CaSO_4_, 175 μM KH_2_PO_4_, 25 μM (NH_4_)H_2_PO_4_, 0.05 μM NaMoO_4_, 2.5 μM H_3_BO_3_, 0.2 μM MnSO_4_, 0.2 μM ZnSO_4_, 0.05 μM CuSO_4_, and 2 μM Fe-EDTA, pH adjusted to 6.0 with KOH. In order to induce ${\mathrm{NO}}_{3}^{–}$ uptake, Ca(NO_3_)_2_ with a final concentration of 0.25 mM was added to the NS for ${\mathrm{NO}}_{3}^{–}$ treated samples (${\mathrm{NO}}_{3}^{–}$). Control samples were obtained growing seedlings for the same times in the NS without Ca(NO_3_)_2_ and adding CaSO_4_ (0.25 mM) in order to balance Ca^2+^. Plants were analyzed for their ability to take up ^15^${\mathrm{NO}}_{3}^{–}$ in the 24 h (0, 4, 8, 15, and 24 h) following both ${\mathrm{NO}}_{3}^{–}$-treated and control samples. At the same times, roots of both ${\mathrm{NO}}_{3}^{–}$-treated and control samples were frozen using liquid nitrogen and stored at -80°C. Three independent growth experiments were performed (three biological replicates). Each sample of each biological replicate was a pool of roots of eight plants.

### ${\mathrm{NO}}_{3}^{–}$ Uptake

Maize seedlings were washed in a 0.1 mM CaSO_4_ solution for 1 min before transferring them to an aerated uptake solution for 5 min. The uptake solution composition is the following: 100 μM Ca(^15^NO_3_)_2_ (98 atom% ^15^N) in 1 mM MES-BTP, pH 6.0. The plants were then washed for 1 min in 0.1 mM CaSO_4_ solution. Cut roots were then dried at 70°C for 48 h and powdered. For each sample, the ^15^N content was determined after isotope ratio mass spectrometry analysis coupled with an elemental analyzer (Delta V IRMS, Thermos Scientific Inc., Waltham, MA, USA).

### PM Vesicles Isolation

Microsome isolation was performed as previously described by Giannini et al. (1988) modifying the composition of the homogenisation buffer (Fischer-Schliebs et al., 1994). Five grams of maize roots were cut and homogenized into 15 mL of ice-cold homogenisation buffer with the following composition: 250 mM sucrose, 2 mM MgSO_4_, 25 mM BTP, 10 mM rac-glycerol 1-phosphate disodium salt hydrate, 2 mM ethylene glycol-bis(*b*-aminoethyl ether)-*N, N, N′, N′*-tetraacetic acid (EGTA), 2 mM ethylenediaminetetraacetic acid (EDTA), 10% (w/v) glycerol, 6% (w/v) choline-iodide, 1 mM phenylmethanesulfonyl fluoride (PMSF), 2 mM sodium- ATP, 2 mM dithiothreitol (DTT), 20 mg/ml chymostatin and 1% (w/v) polyvinylpyrrolidone, titrated to pH 7.6 with MES. The homogenate was filtered through a four-layer gauze and the obtained suspension was subjected to a brief centrifugation in order to separate the soluble part from the cell debris. The recovered supernatant was centrifuged for 25 min at 13000 *g* and the resulting pellet was suspended in homogenization buffer without polyvinylpyrrolidone. The suspension was loaded onto a discontinuous gradient, produced layering a 25% sucrose solution over a 38% sucrose solution. The composition of both sucrose solutions is the following: 2 mM MgSO_4_, 25 mM BTP, 10 mM rac-glycerol 1-phosphate disodium salt hydrate, 2 mM EGTA, 6% (w/v) choline-iodide, 1 mM PMSF, 2 mM sodium-ATP, 2 mM DTT, and 20 mg/ml chymostatin, titrated to pH 7.4 with MES. The gradient was centrifuged for 1 h at 13000 *g*. At the end, microsomal fraction was recovered at the interface of the two sucrose solutions, washed and resuspended in resuspension buffer with the following composition: 20% (w/v) glycerol, 2 mM BTP, 2 mM EDTA, 2 mM EGTA, 1 mM PMSF, 0.5 mM sodium-ATP, 2 mM DTT, and 50 mg/ml chymostatin, titrated to pH 7.0 with MES.

The protein concentration was evaluated by the Bradford method (Bradford, 1976), using BSA as standard after membrane solubilisation adding 0.5 M NaOH to the suspension (Gogstad and Krutnes, 1982).

### Adenosine Triphosphatease Enzyme Activity

The activity was characterized through the determination of the release of Pi as described by Forbush (1983). The assay was performed incubating microsome samples in 0.6 mL of reaction mixture (50 mM Mes-BTP (pH 6.5), 5 mM MgSO_4_, 0.6 mM Na_2_MoO_4_, 5 mM ATP-BTP, and 0.01% Brij 58; Sigma-Aldrich Co. LLC, St Louis, MO, USA) for 30 min at 38°C. Then, 1.5 mM NaN_3_ and 100 mM KNO_3_ were added to all samples in order to inhibit the activity of mitochondrial and tonoplast ATPases, respectively. The reaction was stopped with 1 mL of a stop solution (0.6 M HCl, 3% SDS, 3% (w/v) ascorbic acid and 0.5% (w/v) ammonium molybdate) and incubation on ice for 10 min. Samples were then incubated for 10 min at 38°C after the addition of 1.5 mL of a solution containing 2% (v/v) acetic acid, 2% (w/v) sodium citrate and 2% (w/v) sodium arsenite. The absorbance was measured at 705 nm and the ATPase activity was expressed as nmol of Pi produced per mg protein per h.

### Western Blot Analysis

Protein samples were prepared by suspending microsome fractions corresponding to 10 μg protein in an equal volume of sample buffer. Two different sample buffers were used. For the PM-ATPase analysis, the following buffer was used: 0.125 M Tris- HCl, pH 6.8, 10% (w/v) SDS, 0.2 M DTT, 10% (w/v) glycerol, 500 mg/ml chymostatin, 5 M PMSF, and 0.01% (w/v) bromophenol blue. The resulting mixture was treated at 37°C for 20 min. For the analysis of NRT2 and NRT3, a second buffer was used (0.05 M Tris-HCl, pH 6.8, 4% (w/v) SDS, 12% (w/v) glycerol, 2% (v/v) 2- mercaptoethanol, and 0.05% (w/v) bromophenol blue), and samples were boiled for 5 min. Five micrograms of ECL^TM^ Plex Fluorescent Rainbow Markers (GE Healthcare Amersham^TM^) were used for each SDS-PAGE analysis.

The proteins were separated by a 10% SDS polyacrylamide gel electrophoresis for the analysis of both PM-ATPase and NRT2, and by a 12% SDS polyacrylamide gel electrophoresis for the analysis of NRT3. The proteins were then electroblotted onto a nitrocellulose membrane (Hybond-ECL; GE Healthcare Life Science, Little Chalfont, UK). The obtained membranes were differently used for immunodetection with a polyclonal primary antibody raised against the PM H^+^-ATPase (Agrisera AB, Vännäs, Sweden) and polyclonal primary antibodies against ZmNRT2.1 and ZmNRT3.1A (ZmNAR2.1) produced in collaboration with Agrisera (Agrisera AB, Vännäs, Sweden). The anti-NRT2.1 antibody was raised in rabbit against the following synthetic peptides: (C)EHKAKSVRLFSVANPH, (C)KDSFSKVMWYAVINYR, (C)KGLHSASLKFAENSR. The anti-NRT3.1A antibody was raised in rabbit against the synthetic peptide (C)LDVTTSAKPGQ. Reactive proteins were detected with secondary antibody, peroxidase-conjugated goat anti-rabbit IgG (Sigma-Aldrich Co. LLC). Chemiluminescent signals were produced using the ECL Plus Western Blotting Detection Kit (Amersham), and detected by ChemiDoc^TM^ XRS+ system (Bio-Rad, Hercules, CA, USA). In order to confirm the specificity of the polyclonal primary antibodies against ZmNRT2.1 and ZmNRT3.1A (ZmNAR2.1), the two SDS-PAGE bands were individually analyzed by tandem mass spectrometry as described below. ZmNRT2.1 was identified at about 50 kDa and ZmNRT3.1A (ZmNAR2.1) was identified at about 21 kDa (Supplementary File 1).

### Non-denaturing Deriphat-PAGE

As “stained” molecular weight marker, we used chlorophyll-binding complexes purified from photosynthetic membranes of *Arabidopsis thaliana*. Unstacked thylakoids were isolated from wild type leaves as previously described (Casazza et al., 2001). Presence of a broad-range protease inhibitor cocktail allowed preserving proteome integrity, thereby excluding that supercomplexes identified originate from differential cleavage by processive peptidases. Pigments were extracted from thylakoids with 80% acetone buffered with Na_2_CO_3_, and quantified as previously reported (Croce et al., 2000).

For bidimensional native-/SDS-PAGE separation of supercomplexes, non-denaturing Deriphat-PAGE (1st dimension) was performed following the method developed by (Peter et al., 1991) with modification described in (Havaux et al., 2004). Thylakoids concentrated at 1 mg/ml chlorophylls, and microsome fraction with protein concentration of 100 μg/ml in resuspension buffer (12 mM Tris pH 8.3, 96 mM glycine, 50% v/v glycerol) were solubilised with either 0.8% *n*-dodecyl β-D-maltoside (β-DM) or 0.8% *n*-dodecyl α-D-maltoside (α-DM); 25 μg of chlorophyll (thylakoids) and a volume of microsome fraction corresponding to 50 μg of proteins for each sample were loaded in each lane. For the non-denaturing Deriphat-PAGE experiment performed in order to identify a NRT2/NRT3 complex, sample was solubilized using ß-DM whilst α-DM was used for preparation of samples for the bidimensional native-/SDS-PAGE separation of PM H^+^-ATPase supercomplexes.

For each sample, gel strips from the 1st dimension native-PAGE, corresponding to a range of molecular weight in between ∼100 kDa (trimeric LHCII) and ∼620 kDa (C2S2M2 supercomplex, see, Caffarri et al., 2009), were excised and incubated with gentle shaking in a denaturing buffer (100 mM Tris, 100 mM Tricine, 1 mM EDTA, 6 M urea, 20% v/v glycerol, 4.3% w/v SDS, 5% v/v β - mercaptoethanol) for 1 h at RT. Then, strips were transferred to the top of the SDS/PAGE gel and sealed with 0.5% agarose in running buffer (100 mM Tris, 100 mM Tricine, 1 mM EDTA). SDS-PAGE analysis (2-dimensional separation) was performed with the Tris-Tricine buffer system (Shägger and von Jagow, 1987). In the resolving gel, an acrylamide (48% acrylamide /1.5% bisacrylamide) gradient from 12 to 16% (w/v) was stabilized by a glycerol gradient from 8 to 16%. After electrophoresis, the proteins of interest were visualized by western blot using polyclonal antibody against PM H^+^-ATPase as previously described.

### Protein Identification by Mass Spectrometry

The bands excised from the non-denaturing Deriphat-PAGE were individually analyzed by tandem mass spectrometry (nLC-nESI-MS/MS) using an 6520 Q-TOF mass spectrometer with HPLC Chip Cube source driven by 1200 series nano/capillary LC system (Agilent Technologies) as previously described (Prinsi and Espen, 2015). The spectra interpretation was performed by Spectrum Mill MS Proteomics Workbench (Rev B.04.00.127; Agilent Technologies). Cysteine carbamidomethylation and methionine oxidation were set as fixed and variable modifications, respectively, accepting two missed cleavages per peptide. The search was conducted against the subset of *Zea mays* protein sequences (ID tax: 4577, Oct 2015, *212069 entries*) downloaded from the National Center for Biotechnology Information^1^ and oncatenated with the reverse one. The threshold used for peptide identification was Spectrum Mill score ≥9, Score Peak Intensity ≥50%, mass MH+ Error ≤±10 ppm, Database Fwd-Rev Score ≥2, and Local False Discovery Rate ≤5%. Protein identification was accepted if confirmed by at least two distinct peptides.

### Bioinformatic Analysis

Putative maize PM H^+^-ATPases were primarily identified on the basis of amino acid sequence similarity with the PM H^+^-ATPase of *Nicotiana plumbaginifolia* Viv., *Oryza sativa* L., *Arabidopsis thaliana* (L.) Heynh. (Arango et al., 2003), *Vitis vinifera* (Pii et al., 2014), and *Fragaria vesca* (Valentinuzzi et al., 2015). The amino acid sequences were obtained from public databases^1,^^2^^,^^3^ (The Arabidopsis Information Resource (TAIR), MSU) and the relative accession numbers were reported in Supplementary Table S1. Putative maize PM H^+^-ATPases were identified through BLASTP (Altschul et al., 1997) research using the MaizeSequence.org^4^. BLASTP analysis was carried out using each known protein and selecting the putative maize proteins on the basis of the highest sequence homology value (≥80%). A phylogenetic analysis was carried out using the selected maize proteins encoding for putative members of PM H^+^-ATPase family. Protein sequences of the previously mentioned dicot and monocot plant species were aligned by the ClustalW ver. 2.1 algorithm^5^. Phylogenetic tree was produced using the Phylogenetic Interference Package program^6^ (PHYLIP; University of Washington) and visualized by the FigTree ver. 1.4.2 software^7^.

### Real-Time RT-PCR

Total RNA was extracted from the same root samples used for microsome preparation with the Spectrum Plant Total RNA Kit (Sigma-Aldrich Co. LLC) according to the operating manual. One milligram of total RNA was subjected to DNAse digestion with 10 U of DNAse RQ1, then cDNA was synthesized using the ImProm-II Reverse Transcription System (Promega, Madison, WI, USA). The quality of total RNA and cDNA was checked through a PCR using couples of primers specific for housekeeping genes.

Gene-specific primers were designed for the target genes as well as for the housekeeping genes (see Supplementary Table S2). Real-time reverse transcription-PCR (RT-PCR) experiments were carried out in biological triplicates. The reactions were performed by using the SsoFast EvaGreen Supermix (Bio-Rad) and the Bio-Rad iCycler MyiQ real-time PCR system (Bio-Rad) with the following thermal profile: 95°C for 30 s and 40 cycles at 95°C for 10 s and 60°C for 20 s. The specificity of PCR products was evaluated though the analysis of melting curve and sequencing. The amplification efficiency was calculated from raw data using LinRegPCR software ^8^ (Heart Failure Research Center, Amsterdam, The Netherlands; Ramakers et al., 2003). Two housekeeping transcripts were considered, encoding a putative translation elongation factor Tu family protein isoform 1 (GRMZM2G153541_T01) and a polyubiquitin containing 7 ubiquitin monomers (GRMZM2G118637_T01), respectively. For each transcript, two mean normalized expression values (MNE; Simon, 2003) were calculated using separately the two housekeeping transcripts. A final mean normalized expression value was calculated using a geometric mean of the two normalized expression value obtained for each transcript (Vandesompele et al., 2002).

### Statistical Analysis

Figures report mean values ± SE. Statistical analyses were performed using a Student’s *t*-test.

## Results

### ${\mathrm{NO}}_{3}^{–}$ Uptake and ATP Hydrolysing Activity

**FIGURE 1:**
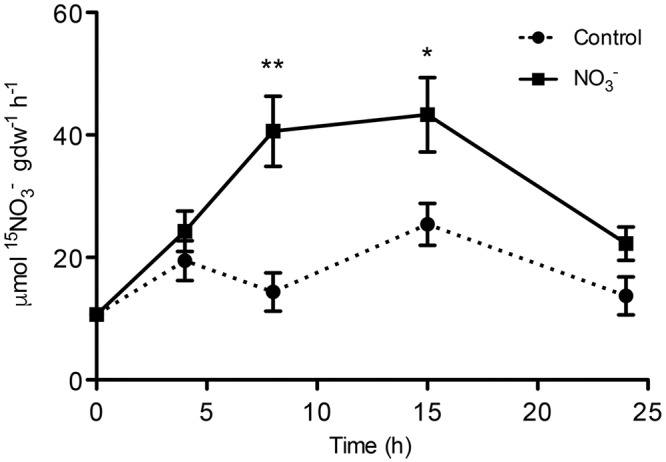
**High-affinity ${\mathrm{NO}}_{3}^{–}$ uptake rate in the roots of maize plants.** ^15^${\mathrm{NO}}_{3}^{–}$ uptake rate by maize plants was measured after different period of treatment (induction) with the anion. At the indicated times, seedlings were transferred to 200 μM ^15^${\mathrm{NO}}_{3}^{–}$ solution and the ${\mathrm{NO}}_{3}^{–}$ uptake was carried out for up to 5 min. Data are the means ± SE; *n* = 3. The statistical significance was determined by means of Student’s *t*-test. (^∗∗^*P* < 0.01; ^∗^*P* < 0.05).

Maize plants cope with the rapid changes in ${\mathrm{NO}}_{3}^{–}$ concentration in soil solution through an uptake system that increases its activity after the exposure to the anion. Our results confirm the existence of this phenomenon, called induction, which has already been described in maize (Quaggiotti et al., 2003; Santi et al., 2003; Zamboni et al., 2014). The induction with 500 μM ${\mathrm{NO}}_{3}^{–}$ caused an increase in anion uptake rate of about four times in roots of treated plants as compared to control plants after 8 h (**Figure 1**). The ${\mathrm{NO}}_{3}^{–}$ uptake rate declined after 15 h in roots of both treated and control plants (**Figure 1**).

**FIGURE 2:**
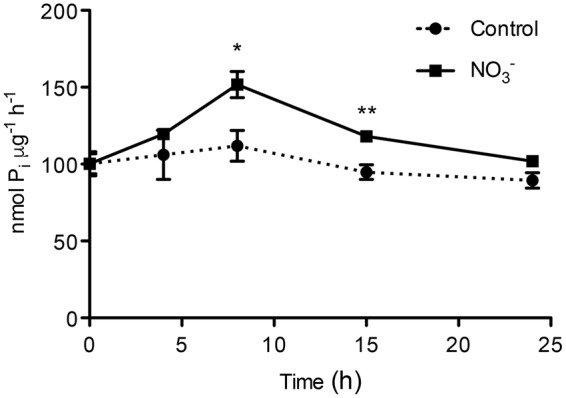
**ATP hydrolysing activity in plasma membrane-enriched vesicles from the roots of maize plants.** ATP hydrolysing activity was measured in plasma membrane (PM)-enriched vesicles isolated from both ${\mathrm{NO}}_{3}^{–}$-induced roots and in control roots. Data are the means ± SE; *n* = 3. The statistical significance was determined by means of Student’s *t*-test. (^∗∗^*P* < 0.01; ^∗^*P* < 0.05).

The ATP hydrolysing activity of roots microsomes fraction was determined during the experiment (0-24 h) for both ${\mathrm{NO}}_{3}^{–}$-treated and control plants (**Figure 2**). After 8 h of ${\mathrm{NO}}_{3}^{–}$ induction, we observed a statistically significant increase in the activity of the PM H^+^-ATPase of about 1.5-fold as compared to control roots at the starting point (0 h). The rate of ATP hydrolysis declined afterwards, reaching the initial value at 24 h. The use of the specific PM H^+^-ATPase inhibitor V_2_O_5_ (0.1 mM) caused an average inhibition of the enzyme activity of about 90% (data not shown), demonstrating that the microsomal preparation was enriched in PM vesicles (Gallagher and Leonard, 1982; Brauer et al., 1989).

### Protein Levels of ${\mathrm{NO}}_{3}^{–}$ Uptake System

**FIGURE 3:**
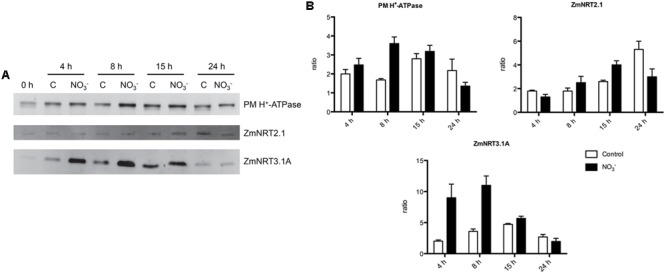
**Western blot analysis carried out on the microsomal fraction extracted from the root tissue of maize seedlings treated with ${\mathrm{NO}}_{3}^{–}$ for the indicated times. (A)** Western blot analysis performed using anti-PM H^+^-ATPase (upper panel), anti-NRT2.1 (middle panel) or anti-NRT3.1A (lower panel) antibodies. The same amount of proteins was loaded in each lane. The protein molecular weights were estimated using the ECL^TM^ Plex Fluorescent Rainbow Markers (GE Healthcare Life Sciences). **(B)** Densitometric analysis of PM H^+^-ATPase, NRT2.1, and NRT3.1A. Data are expressed as ratio of the corresponding 0 h sample (signals were quantified using Quantity One software, Bio-Rad). Data are the means ± SE, *n* = 3.

Changes in protein levels of the two-components system of high-affinity ${\mathrm{NO}}_{3}^{–}$ transport (i.e., ZmNRT2.1 and ZmNRT3.1A) and PM H^+^-ATPase were characterized through western blot analysis performed on the microsomal preparation. Roots of both ${\mathrm{NO}}_{3}^{–}$ -induced and non-induced maize plants have been sampled at 0, 4, 8, 15, and 24 h (**Figure 3A**). Densitometric analyses were carried out to determine the abundance of ZmATPase, by an anti-PM H^+^-ATPase antibody that yielded a single band with an apparent molecular weight of about 100 kDa (data not shown). The protein levels were higher in ${\mathrm{NO}}_{3}^{–}$-treated roots than control ones, in particular after 8 h of induction (**Figure 3B**). In order to study the changes in levels of proteins belonging to the NRT2/NRT3 system involved in the high affinity transport of ${\mathrm{NO}}_{3}^{–}$ in plant roots (Nacry et al., 2013), antibodies directed towards ZmNRT2.1 and ZmNRT3.1A were developed in this work. Western blots performed with the anti-NRT2.1 and the anti-NRT3.1A antibodies detected a polypeptide of ∼ 50 and ∼ 21 kDa, respectively (data not shown). ${\mathrm{NO}}_{3}^{–}$ caused a weak increase in ZmNRT2.1 protein levels in treated roots relative to the control ones; nevertheless the higher increase was at 15 h after treatment (**Figure 3**). However, the treatment with the anion caused a stronger effect on ZmNRT3.1A proteins, which showed the highest abundance between 4 and 15 h with a peak after 8 h (**Figure 3**).

### Identification of PM H^+^-ATPase Genes in Maize Genome

**FIGURE 4:**
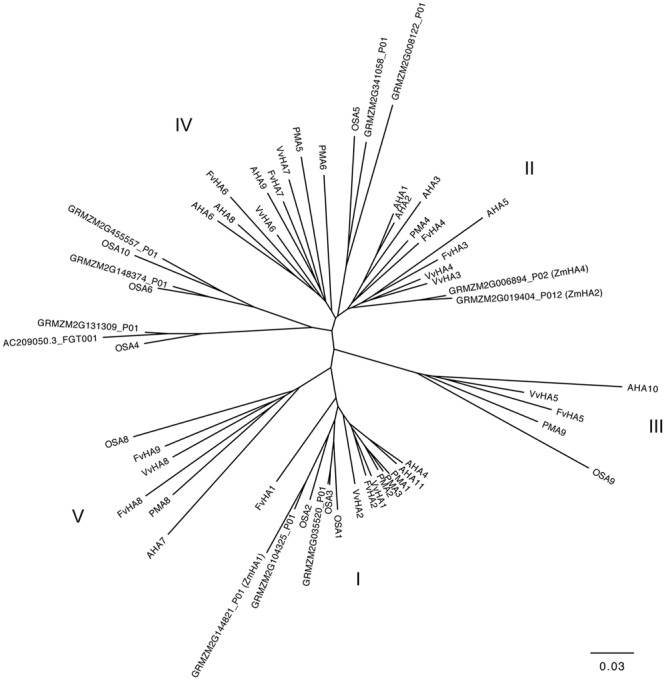
**Phylogenetic tree showing the relationships between the PM H^+^-ATPase from *Zea mays, Vitis vinifera, Fragaria vesca, Arabidopsis thaliana, Nicotiana plumbaginifolia* and *Oryza sativa*.** Phylogenetic tree was built using the Phylogenetic Interference Package program (PHYLIP) and it was visualized using FigTree ver. 1.4.2 software (for protein ID codes, see Supplementary Table S1). Bootstrap values from 1000 replicates were used to estimate the confidence limits of the nodes. The scale bar represents a 0.03 estimated amino acid substitutions per residue.

The PM H^+^-ATPases are encoded in plants by genes belonging to a subfamily of P-type ATPase family, the P3-subfamily (Pedersen et al., 2012; Lang et al., 2014). Twelve and nine members of ATPase P3-subfamily were identified in *Arabidopsis thaliana* and in *Nicotiana plumbaginifolia* genome, respectively (Palmgren, 2001). In addition, it has been also recently reported that rice genome and grapevine genome encode for eight potential H^+^-ATPase genes (Pii et al., 2014; Wang et al., 2014), whilst *Fragaria vesca* genome contains nine putative H^+^-ATPase genes (Valentinuzzi et al., 2015). Lang et al. (2014) reported that in maize genome are present 10 genes encoding PM H^+^-ATPase. Here we report results of a Blastp analysis performed using the datasets of proteins predicted from maize genome ^9^. We identified 11 maize transcripts encoding putative PM H^+^-ATPase (**Figure 4**) distribute in three of the five subfamilies of the ATPase P3-subfamily (Arango et al., 2003). Three putative maize PM H^+^-ATPase belong to the subfamily I (GRMZM2G035520_P01, GRMZM2G104325_P01, and GRMZM2G144821_P01), four to subfamily II (GRMZM2G019404_P01 GRMZM2G006894_P01, GRMZM2G008122_P01 and GRMZM2G341058_P01) and four to subfamily IV (AC209050.3_FGT001, GRMZM2G131309_P01, GRMZM2G148374_P01 and GRMZM2G455557_P01) (**Figure 4**).

### Gene Expression Analysis

**FIGURE 5:**
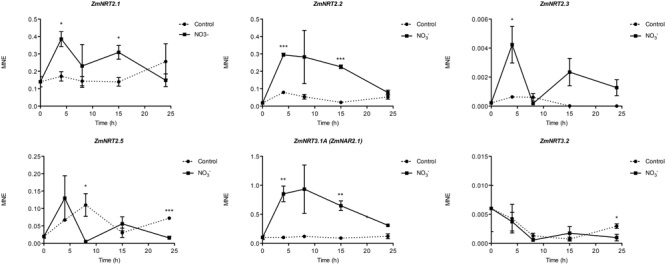
**Time course expression analysis of *NRT2* and *NRT3* genes in ${\mathrm{NO}}_{3}^{–}$-induced maize roots.** The expression levels of *ZmNRT2.1, ZmNRT2.2, ZmNRT2.3, ZmNRT2.5, ZmNRT3.1A*, and *ZmNRT3.1B* were assessed by qRT-PCR in maize roots treated for the indicated times with ${\mathrm{NO}}_{3}^{–}$. The data were normalized to two internal controls, elongation factor 1-alpha (GRMZM2G153541_T01) and polyubiquitin containing seven ubiquitin monomers (GRMZM2G118637_T01). The relative expression ratios were calculated using untreated control roots as a calibrator sample. The values reported are means ± SE; *n* = 3 (^∗^*P* < 0.05; ^∗∗^*P* < 0.01; ^∗∗∗^*P* < 0.001).

**FIGURE 6:**
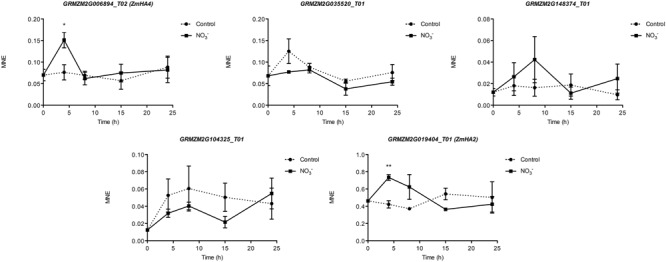
**Time course expression analysis of *PM H^+^-ATPase* genes in ${\mathrm{NO}}_{3}^{–}$-induced maize roots.** The expression levels of GRMZM2G006894_T01 (*ZmHA4*), GRMZM2G035520_T01, GRMZM2G148374_T01, GRMZM2G104325_T01 and GRMZM2G019404_T01 (*ZmHA2*) were assessed by qRT–PCR in maize roots treated for the indicated times with ${\mathrm{NO}}_{3}^{–}$. The data were normalized to two internal controls, elongation factor 1-alpha (GRMZM2G153541_T01) and polyubiquitin containing seven ubiquitin monomers (GRMZM2G118637_T01). The relative expression ratios were calculated using untreated control roots as a calibrator sample. The values reported are means ± SE; *n* = 3 (^∗^*P* < 0.05; ^∗∗^*P* < 0.01; ^∗∗∗^*P* < 0.001).

Gene expression analyses were performed in order to identify which maize transcripts encoding members of the NRT2, NRT3, and PM H^+^-ATPase gene families could be mainly involved in biochemical events triggered by ${\mathrm{NO}}_{3}^{–}$ treatment (500 μM). The quantifications of gene expression have been carried out at each time point considered in the experiment (0, 4, 8, 15, and 24 h) (**Figures 5** and **6**). Four NRT2 and three NRT3 transcripts were previously identified in maize genome (Plett et al., 2010). Concerning the NRT2 family, we observed higher expression levels for *ZmNRT2.1* (GRMZM2G010280_T01) and *ZmNRT2.2* (GRMZM2G010251_T01) with a peak after 4 h of treatment (**Figure 5**). In the case of both genes, the expression levels were maintained significantly higher in the successive time points in comparison to those detected in control roots, and declined at 24 h of treatment. Despite having a significantly lower expression level as compared to *ZmNRT2.1* and *ZmNRT2.2, ZmNRT2.3* (GRMZM2G163866_T01) showed a statistically significant transcriptional induction at 4 h after ${\mathrm{NO}}_{3}^{–}$ treatment with respect to control samples (**Figure 5**). In the case of NRT3 family, the *ZmNRT3.1A* (GRMZM2G179294_T01) was the member showing the highest expression in the root tissue of both ${\mathrm{NO}}_{3}^{–}$ treated and untreated plants. It also displayed the greater transcriptional response to the induction relative to control plants (**Figure 5**). Only five out of 11 putative PM H^+^-ATPase encoded in the maize genome resulted expressed in roots in our experimental conditions (**Figure 6**). The expression profiling in treated and control roots of GRMZM2G019404_T01 (*ZmHA2*) putative PM H^+^-ATPase suggested that the transcript might be involved in the induction phenomenon. In fact, it showed the highest expression levels in roots and displayed a statistically significant induction in response to ${\mathrm{NO}}_{3}^{–}$ treatment after 4 h (**Figure 6**). These observations were in line with the profile detected for both ${\mathrm{NO}}_{3}^{–}$ uptake (**Figure 1**) and ATPase hydrolysing activity (**Figure 2**). Similar role could be attributed to the GRMZM2G006894_T02 (*ZmHA4*) on the basis of its transcriptional profile in treated and control roots, despite its lower levels (**Figure 6**). The transcripts GRMZM2G035520_T01, GRMZM2G148374_T01 and GRMZM2G104325_T01 did not seem involved in the induction phenomenon, given their low expression in both treated and control roots (**Figure 6**).

### Analysis of Protein Complexes

Interactions between members of NRT2 family with NRT3 (NAR2) proteins were reported in *Arabidopsis* (Orsel et al., 2006; Yong et al., 2010; Kotur et al., 2012; Kotur and Glass, 2015), rice (Yan et al., 2011; Liu et al., 2014), barley (Ishikawa et al., 2009), and *Chrysanthemum morifolium* (Gu et al., 2016). These proteins appear to be associated in an oligomer of ∼150 kDa as observed in *Arabidopsis* roots in response to ${\mathrm{NO}}_{3}^{–}$ induction, thus suggesting that iHATS relies on a tetrameric aggregation of two subunits, namely AtNRT2.1 and AtNAR2.1 (Yong et al., 2010). Recently, a 150 kDa complex of AtNRT2.5 and AtNAR2.1 was shown to be involved in constitutive HATS (cHATS; Kotur and Glass, 2015).

In order to analyze the organization of HATS components (NRT2 and NRT3) during ${\mathrm{NO}}_{3}^{–}$ induction in maize, a nondenaturing PAGE was carried out using the microsomal fraction isolated from ${\mathrm{NO}}_{3}^{–}$-treated roots, sampled at 8 h and solubilized with 0.8% n-dodecyl ß-D-maltoside (ß-DM). Chlorophyll-binding complexes from *Arabidopsis thaliana* thylakoids were used as a native molecular weight marker (Supplementary Figure S1). On the basis of previous evidence from *Arabidopsis thaliana* (Yong et al., 2010; Kotur and Glass, 2015), a tight gel slice in the region of ∼ 150 kDa was excised and analyzed by nLC-nESI-MS/MS. The co-presence of ZmNRT2.1 (AAN05088.1; GRMZM2G010280_P01) and ZmNRT3.1 (ZmNAR2.1; NP_001105929.1; GRMZM2G179294_T01) proteins was confirmed (Supplementary File 1), thus suggesting that an oligomer composed by two ZmNRT2.1 and two ZmNRT3.1A might be involved in the ${\mathrm{NO}}_{3}^{–}$ uptake in maize roots upon induction.

**FIGURE 7:**
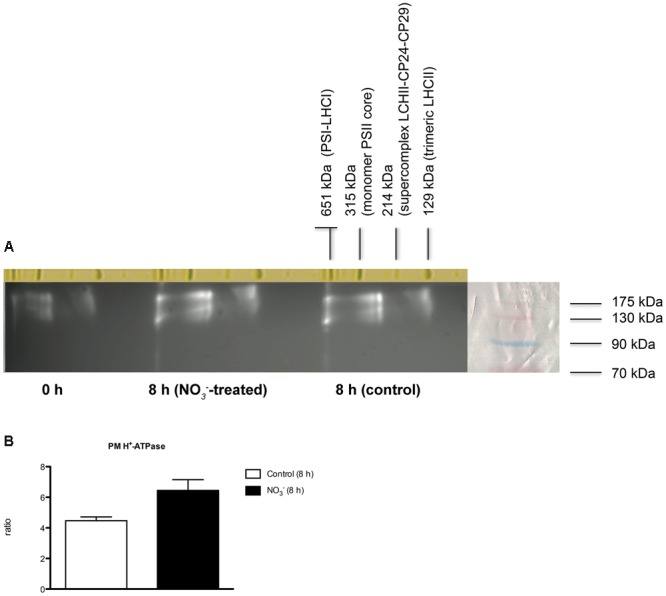
**Separation of PM H^+^-ATPase complexes by non-denaturing Deriphat-PAGE followed by SDS-PAGE. (A)** Western blot analysis performed using antibodies anti-PM H^+^-ATPase. The molecular weight of each protein marker (ECL Plex Fluorescent Rainbow Markers) was reported on the right side. **(B)** Densitometric analysis of PM H^+^-ATPase. Data are expressed as ratio of corresponding 0 h sample considering the sum of all three forms (signals were quantified using PDQuest^TM^ 2-D Analysis Software, Bio-Rad). Data are the means ± SE, *n* = 3.

The organization of PM H^+^-ATPase into supramolecular complexes was analyzed by a bidimensional native-/SDS-PAGE. Microsomal fractions, isolated from maize roots sampled at 0 and 8 h (for both treated and control plants), were solubilized with 0.8% *n*-dodecyl α-D-maltoside (α-DM) (**Figure 7**) and protein complexes separated by non-denaturing PAGE; chlorophyll-binding complexes from *Arabidopsis thaliana* thylakoids were loaded on the first separation stage, as a native molecular weight marker. The 100-700 kDa regions from the first dimension were excised and further fractionated by denaturing SDS-PAGE in a second dimension, and the 2D map was analyzed by immunoblotting (**Figure 7A**). The anti-PM H^+^-ATPase antibody detected three protein spots at ∼100 kDa in the 2D map, namely a molecular weight consistent with former results (**Figure 3A**). Interestingly, western blot on 2D map revealed the presence of three oligomeric states of PM H^+^-ATPase *in vivo*, whose apparent molecular masses were ∼120, 240, and 700 kDa, respectively (**Figure 7A**). Densitometric analysis of all three forms performed for each sample showed that the protein abundance followed the order: 0 h <8 h control <8 h ${\mathrm{NO}}_{3}^{–}$-treated (**Figure 7B**). The bands corresponding to PM H^+^-ATPase complexes were excised and analyzed by tandem nLC-nESI-MS/MS, which confirmed the presence of ZmMHA2 subunit (NP_001292776.1; GRMZM2G019404_P01) in each of the three bands (Supplementary File 1).

## Discussion

Nitrate is the main source of mineral N for plants in well-aerated soils. Physiological and molecular bases of ${\mathrm{NO}}_{3}^{–}$ uptake of plant roots are known, in particular for herbaceous species (Nacry et al., 2013). Nonetheless, the regulatory mechanism controlling the uptake systems is not fully elucidated yet due to lack of knowledge of the process at all the regulatory levels (e.g., ${\mathrm{NO}}_{3}^{–}$ uptake and PM H^+^-ATP activity, protein and transcript levels). However, some transcription factors and transcriptional mechanisms controlling gene expression during changes in ${\mathrm{NO}}_{3}^{–}$ availability have been identified in the model plant *Arabidopsis thaliana* (Vidal et al., 2015). The phenomenon of “induction” consists in an increase in ${\mathrm{NO}}_{3}^{–}$ uptake rate by roots as a consequence of the exposure to the anion and involves the activity of inducible high-affinity transport system (iHATS) (Siddiqi et al., 1989); iHATS has already been described in several plant species (Kronzucker et al., 1995; Min et al., 1998; Okamoto et al., 2003; Santi et al., 2003; Pii et al., 2014). The induction has been already studied in maize roots focusing on transcriptional changes of ${\mathrm{NO}}_{3}^{–}$ uptake systems and at protein level limited to PM H^+^-ATPase (Santi et al., 2003); recently, the induction phenomenon has also been studied at whole transcriptome level in roots of two inbreed lines featuring different nitrogen use efficiency (NUE) (Zamboni et al., 2014). The availability of maize genome sequence (Schnable et al., 2009) allowed the identification of all putative members of the gene families involved in ${\mathrm{NO}}_{3}^{–}$ uptake system (e.g., NRT2 and NRT3; Plett et al., 2010) and members of the PM H^+^-ATPase (**Figure 4**). This information gives a complete picture of the molecular entities playing a role in the ${\mathrm{NO}}_{3}^{–}$ uptake systems and also allows investigating how each component can vary during changes in ${\mathrm{NO}}_{3}^{–}$ availability. In this work we characterized changes in ${\mathrm{NO}}_{3}^{–}$ uptake rate, ATP hydrolysing activity, changes in protein and transcriptional levels during the anion treatment (0-24 h) comparing ${\mathrm{NO}}_{3}^{–}$ treated vs. control roots. In a previous work, Santi et al. (2003) found that ${\mathrm{NO}}_{3}^{–}$ uptake rate in maize plants (cv. Cecilia) displayed a peak of induction around 4 h after onset of the treatment. Yet, also different maize inbreed lines have been characterized for a different response to ${\mathrm{NO}}_{3}^{–}$ supply in term of response to the induction, which could be related to NUE (Zamboni et al., 2014). Our data showed that the uptake rate, measured through ^15^N, had an induction peak between 8 and 15 h after the anion treatment (**Figure 1**), thus confirming the phenomenon is genotype-specific. Furthermore, we observed significant differences in the uptake rate between treated and control samples, as well as ATP hydrolysing activity ratio, the latter showing a similar profile to that of ${\mathrm{NO}}_{3}^{–}$ uptake rate (**Figure 2**).

Concerning the abundance of the components thought to be involved in the whole mechanism of ${\mathrm{NO}}_{3}^{–}$ uptake, the main differences were observed at 15 h after the induction for ZmNRT2.1, whilst the increase in the uptake rate was observed starting from 8 h after the treatment (**Figure 3B**). Interestingly, in the case of ZmNRT3.1A (ZmNAR2.1), we observed a stronger increase in the protein level starting from 4 h after the induction, showing a peak at 8 h and decreasing afterwards (**Figure 3B**) thus correlating with the anion uptake rate profile (**Figure 1**). These results suggest that the quick responses of maize plants to the variation of ${\mathrm{NO}}_{3}^{–}$ concentrations might be mainly due to both transcriptional and translational regulation of the accessory protein ZmNRT3.1A. Similarly, Ishikawa et al. (2009) showed that in response to a 1 mM ${\mathrm{NO}}_{3}^{–}$ treatment, the profile of anion uptake displayed a stronger correlation with the changes in the transcript and protein level of the accessory protein relative to HvNRT2s transporters. In addition our results showed in maize roots a ∼150 kDa oligomer, likely formed by two ZmNRT2.1 and two ZmNRT3.1A, which plays a role in the high affinity transport in response to 8 h of ${\mathrm{NO}}_{3}^{–}$ treatment. Taken together, our results further confirmed that the transporter ZmNRT2.1 and the accessory protein ZmNRT3.1A are the main components of iHATS during the “induction” phenomenon and that the regulation of this transport system is mainly based on both transcriptional and translational regulation of the accessory protein ZmNRT3.1A.

The involvement of some members of PM H^+^-ATPase gene family in the ${\mathrm{NO}}_{3}^{–}$ induction phenomenon has been previously described (Santi et al., 2003; Sorgonà et al., 2011; Pii et al., 2014). The availability of maize genome sequence allowed the identification of 11 putative maize transcripts encoding PM H^+^-ATPase (**Figure 4**) and the phylogenetic analysis demonstrated that the PM H^+^-ATPase are not uniformly distributed within the five subfamilies predicted by Arango et al. (2003) (**Figure 4**). The possible role of the different isoforms of ATPase in nutrients uptake has been discussed (Sondergaard et al., 2004) and the existence of a functional specialization was hypothesized. It has been shown that, in *Arabidopsis thaliana* plants, the isoform 2 (AHA2) is the main PM H^+^-ATPase playing a role in the rhizosphere acidification for iron (Fe) acquisition, whilst the other isoform (AHA1), despite being expressed in the root tissue, is not responsive to Fe fluctuation, thus having only an housekeeping function (Santi and Schmidt, 2009). In our experimental conditions, only five members of this gene family were expressed in roots. According to the gene expression profiles (**Figure 6**), the transcripts GRMZM2G019404_T01 (*ZmMHA2*, previously named *MHA3* by Santi et al. (2003)) resulted the most responsive to ${\mathrm{NO}}_{3}^{–}$ treatment, showing a statistically significant increase at 4 h in ${\mathrm{NO}}_{3}^{–}$-treated roots. However, previous studies, based on semi-quantitative RT-PCR approaches carried out only in treated maize roots, suggested that the most responsive isoforms to ${\mathrm{NO}}_{3}^{–}$ provision were *MHA3* and *MHA4* (GRMZM2G006894_T02) (Santi et al., 2003; Sorgonà et al., 2011). In our experimental conditions, we recorded higher expression levels of the GRMZM2G019404_T01 (*ZmMHA2*) transcripts as compared with GRMZM2G006894_T02 *(ZmMHA4*) and, for both, the expression profiles in ${\mathrm{NO}}_{3}^{–}$-treated roots are in line with the higher ${\mathrm{NO}}_{3}^{–}$ uptake rate and ATP hydrolysing activity observed within the 15 and 8 h, respectively (**Figures 1** and **2**). In addition, the increases in abundance of PM H^+^-ATPase transcripts (**Figure 6**) during the first 8 h of ${\mathrm{NO}}_{3}^{–}$ treatment are in good agreement with the changes in protein amount observed in the same conditions (**Figure 3**). The increase in the PM H^+^-ATPase protein levels during the induction was also confirmed by the analysis of oligomeric complexes (**Figure 7B**; Supplementary File 1). This analysis showed that PM H^+^-ATPase migrates as multiple bands with different apparent masses (Supplementary File 1), containing undissociated H^+^-ATPase dimers and hexamers (**Figure 7A**). Accordingly, two transcripts encoding this protein resulted significantly affected by ${\mathrm{NO}}_{3}^{–}$ during the induction (**Figure 6**). The results hereby presented highlight that, at least in the case of PM H^+^-ATPase, the ATP hydrolysing activity and the protein abundance data fit only partially with a statistically significant change in transcript levels, thus suggesting post-transcriptional and/or post-translational mechanism might be involved in the control of the enzymatic activity. In fact, several studies have highlighted that also the formation of multimeric complexes could play a role in regulating the PM H^+^-ATPase activity. First structural studies showed assembly of PM H^+^-ATPase into hexameric complexes (Huang and Berry, 1990; Auer et al., 1998), while later works highlighted association of the 14-3-3 proteins to both dimeric and hexameric structure of PM H^+^-ATPase (Briskin and Reynolds-Niesman, 1989; Kanczewska et al., 2005; Ottmann et al., 2007). In particular, Kanczewska et al. (2005) showed that, in tobacco cells transferred to fresh culture medium, the PM H^+^-ATPase activity increased together with protein phosphorylation and the 14-3-3 binding; such protein modifications were hypothesized to be involved in the formation of the PM H^+^-ATPase hexameric complex (Kanczewska et al., 2005).

The functional role of these oligomeric states still awaits elucidation, however, recently Justesen et al. (2013) described the monomeric complex as the active form of PM H^+^-ATPase. We observed an increase in levels of all three states (monomeric, dimeric, and hexameric) with time and with ${\mathrm{NO}}_{3}^{–}$ treatment (**Figure 7B**), suggesting that oligomeric complexes could participate to the induction phenomenon as well.

## Conclusion

Our data provide for the first time a comprehensive picture of the molecular entities (transporters, accessory proteins, and pumps) involved in the enhanced ${\mathrm{NO}}_{3}^{–}$ uptake following the exposure of maize roots to the anion. Results suggest that maize roots cope with ${\mathrm{NO}}_{3}^{–}$ fluctuation in the soil solution by regulating the functionality of the iHATS in the short period, mainly through the modulation at transcriptional/translational level of the accessory protein ZmNRT3.1A. In addition, the iHATS is based on the formation of a ∼150 kDa oligomer of ZmNRT2.1 and ZmNRT3.1A. Furthermore, by means of a nondenaturing Deriphat-PAGE approach, we demonstrated for the first time that PM H^+^-ATPase occurs *in vivo* as hexameric complex, in addition to monomeric and dimeric forms previously described.

## Author Contributions

YP, MA, ZV, and AZ made a substantial contribution to data collection and interpretation and manuscript drafting. LD was responsible for non-denaturing Deriphat-PAGE experiments and KG for IRMS analyses; BP and LE developed the anti-NRT2.1 antibody and were responsible for protein identification by mass spectrometry. AZ and ZV participated in the project’s design and coordination. All the authors critically revised the manuscript.

## Conflict of Interest Statement

The authors declare that the research was conducted in the absence of any commercial or financial relationships that could be construed as a potential conflict of interest.

The reviewer AS and handling Editor declared their shared affiliation, and the handling Editor states that the process nevertheless met the standards of a fair and objective review.

## Footnotes

1 http://www.ncbi.nlm.nih.gov/

2 http://www.uniprot.org/uniprot/

3 maizesequence.org

4 http://ensembl.gramene.org/Zea_mays/Info/Index

5 http://clustalw.ddbj.nig.ac.jp/

6 http://evolution.genetics.washington.edu/phylip.html

7 http://tree.bio.ed.ac.uk/software/figtree/

8 http://www.hartfaalcentrum.nl/index.php?main=files&sub=LinRegPCR

9 http://ensembl.gramene.org/Zea_mays/Info/Index
