# Supplementary material for: Time-Resolved Investigation of Molecular Components Involved in the Induction of NO3– High Affinity Transport System in Maize Roots
Source: Front Plant Sci. 2016 Nov 8;7:1657. doi: 10.3389/fpls.2016.01657 (PMC5099785; doi:10.3389/fpls.2016.01657)
Supplement: Supplementary file 1 [file Table_1.PDF]

Supplementary Table S1

Protein name and Uniprot entry of PM H^+-ATPases of *Nicotiana plumbaginifolia*, *Oryza sativa* L., *Arabidopsis thaliana* (L.) Heynh. (Arango *et al.*, 2003), *Vitis vinifera* (Pii *et al.*, 2014) and Transcript ID for PM H^+-ATPases of *Fragaria vesca* (Valentinuzzi *et al.*, 2015; https://phytozome.jgi.doe.gov/pz/portal.html#!info?alias=Org_Fvesca) used in bioinformatic analyses.

Nicotiana plumbaginifolia		*Arabidopsis thaliana*		*Oryza sativa*		*Vitis vinifera*		*Fragaria vesca*	
**Protein name**	**Entry**	**Protein name**	**Entry**	**Protein name**	**Entry**	**Protein name**	**Entry**	**Protein name**	**Transcript ID**
PMA1	Q08435	AHA1	P20649	OSA1	Q43001	VvHA1	D7SIH5	FvHA1	mrna01281.1-v1.0-hybrid
PMA2	Q42932	AHA2	P19456	OSA2	Q43002	VvHA2	D7TX08	FvHA2	mrna09568.1-v1.0-hybrid
PMA3	Q08436	AHA3	P20431	OSA3	Q9XEL7	VvHA3	F6H3A8	FvHA3	mrna08702.1-v1.0-hybrid
PMA4	Q03194	AHA4	Q9SU58	OSA4	Q8L6I0	VvHA4	D7SQD1	FvHA4	mrna17015.1-v1.0-hybrid
PMA6	Q9SWH2	AHA5	Q9SJB3	OSA5	Q8RW30	VvHA5	F6HXK4	FvHA5	mrna05497.1-v1.0-hybrid
PMA8	Q9SWH1	AHA6	Q9SH76	OSA6	Q8RW29	VvHA6	D7T534	FvHA6	mrna30866.1-v1.0-hybrid
PMA9	Q9SWH0	AHA7	Q9LY32	OSA7	Q7XPY2	VvHA7	A5B4B3	FvHA7	mrna15943.1-v1.0-hybrid
		AHA8	Q9M2A0	OSA8	Q8RW27	VvHA8	D7SLX8	FvHA8	mrna10846.1-v1.0-hybrid
		AHA9	Q42556	OSA9	Q8RW26			FvHA9	mrna04924.1-v1.0-hybrid
		AHA10	Q43128	OSA10	Q8RW25				
		AHA11	Q9LV11						
From Arango *et al.*, 2003						From Pii *et al.*, 2013		From Valentinuzi *et al.*, 2015	

Supplementary Table S2
Sequence of forward and reverse primers used in Real-time RT-PCR experiments.

Transcript_ID (maizesequence.org;release-5b)	Description	Forward primer	Reverse primer
GRMZM2G153541_T01	Elongation factor 1-alpha	5'-TATCTGTCTGGTGCTGTGCT-3'	5'-TCATAGATTACTTGTTCACGC-3'
GRMZM2G118637_T01	Polyubiquitin containing 7 ubiquitin monomers	5'-GCTGCTGTATCTGGGTTATC-3'	5'-CGCACGATAGTTTTGGGTAA-3'
GRMZM2G010280_T01	ZmNRT2.1	5'-CGACGATCACCTATACCTCT-3'	5'-TCATGTCAACGGAGCACACG-3'
GRMZM2G010251_T01	ZmNRT2.2	5'-ATGTTCACCTGCTACCTACC-3'	5'-GAATATCGTTGGCACATCTC-3'
GRMZM2G163866_T01	ZMNRT2.3	5'-TTGCTCGATACTCCTGCTT-3'	5'-GCACAGGAAATACTACGACG-3'
GRMZM2G455124_T01	ZmNRT2.5	5'-AACACACACAAGCATACGGT-3'	5'-CACACAGAAATTACCACACG-3'
GRMZM2G179294_T01	ZmNRT3.1A (ZmNAR2.1)	5'-AGTGGCTGTCGTTGCTGATT-3'	5'-GGTAATTTTGACGCACACAC-3'
GRMZM2G163494_T01	ZmNRT3.1B (ZmNAR2.2)	5'-ACGCGTCATGTTGTGTAGTG-3'	5'-GAATTTTGGTTGACAGGCAC-3'
GRMZM2G008122_T01	PM H+-ATPase	5'-CCTTGCCTCGCTAAGTTGTT-3'	5'-GTACGTAGCTCCCAATTAGG-3'
GRMZM2G019404_T01	PM H+-ATPase (ZmHA2)	5'-AACACCTTTGCTGCCCGAC-3'	5'-GAAACTCCCTAGAAAGACGG-3'
GRMZM2G006894_T02	PM H+-ATPase (ZmHA4)	5'-TGCCACCCTTGTTGTTCTTG-3'	5'-TGTCTCCAATCACATCACCG-3'
GRMZM2G035520_T01	PM H+-ATPase	5'-CGGCGTGTGAATTTGATGGT-3'	5'-GTGAGGAGAGGACAGAAGAA-3'
GRMZM2G341058_T01	PM H+-ATPase	5'-GTTGCTGTACTCATTGGGAA-3'	5'-CGACGACTACTATATCAAGG-3'
GRMZM2G104325_T01	PM H+-ATPase	5'GCGGGAAATGAATGATGGTC-3'	5'-ACAGCATACAAGGGGTGAGT-3'
AC209050.3_FGT001	PM H+-ATPase	5'-TTTGGAGGAGGCGATTTGGA-3'	5'-ACCTAGATATTTCCCTGCTC-3'
GRMZM2G131309_T01	PM H+-ATPase	5-GGAGGAGGAACTGTAGGATT-3'	5'-TGCGACAGATAAACATACCC-3'
GRMZM2G148374_T01	PM H+-ATPase	5'-GCAGACAAATACAAACCACAC-3	5'-CTACAACGACACGCCATGAA-3'
GRMZM2G144821_T01	PM H+-ATPase (ZmHA1)	5'-TAGGGGAAGATGAGGATGGA-3'	5'-AGTTTCTATATTGCTGCCTAG-3'
GRMZM2G455557_T01	PM H+-ATPase	5'-CGTTTATCTGGGTGCTTGTC-3'	5'-TAGAAGGTCGGAGGAGGAG-3'
