# Supplementary material for: Time-Resolved Investigation of Molecular Components Involved in the Induction of NO3– High Affinity Transport System in Maize Roots
Source: Front Plant Sci. 2016 Nov 8;7:1657. doi: 10.3389/fpls.2016.01657 (PMC5099785; doi:10.3389/fpls.2016.01657)
Supplement: Supplementary file 2 [file Data_Sheet_1.PDF]

SUPPLEMENTARY FILE 1: statistical data about protein characterization by nLC-nESI-MS/MS.

Band of ~50 kDa isolated with SDS-PAGE experiment after identification through Western blot analysis performed using the anti-NRT2.1 antibody.

>gi|29412129|gb|AAN05088.1| putative high affinity nitrate transporter [Zea mays]
MAAVGAPGSSLHGVTGREPAFAFSTEHEEAASNGGK**FDLPVDSEHK**AKSVRLFSVANPHMRTFHLSWISFFTCFVSTFAAAPLVPIIRDNLN
LTK**ADIGNAGVASVSGSIFSR**LTMGAVCDLLGPRYGCAFLIMLSAPTVFCMSLIDDAAGYITVRFLIGFSLATFVSCQYWMSTMFSSKIIGTV
NGLAAGWGTMGRRRHAAHMPLVYDVIRKCGATPFTAWRLAYFVPGLMHVVMGVLVLTLGQDLPDGNLRSLQKKGNVNKDSFSKVMW
YAVINYRTWIFVLLYGYCMGVELTTDNVIAEYMYDRFDLDLRVAGTIAACFGMANIVARPMGGIMSDMGARYWGMRARLWNIWILQTA
GGAFCLWLGRASTLPVSVVAMVLFSFCAQAACGAIFGVIPFVSRRSLGIISGMTGAGGNFGAGLTQLLFFTSSTYSTGRGLEYMGIMIMACT
LPVVFVHFPQWGSMFFPPSATADEEGYYASEWNDDEKSKGLHSASLKFAENSRSERGKR**NVIQADAAATPEHV**

Amino acid coverage: 8%.

z	Score	Fwd-Rev Score	SPI (%)	FDR(%) L	FDR(%) G	Sequence Map	Measured m/z (Da)	Matched MH+ (Da)	Error (ppm)
2	**20.13**	20.13	**93.0**	<0.1%	<0.1%	**(R)N V|I|Q/A|D/A/A/A|T|P E/H V(-)**	718.3638	1435.718	1.9
2	**13.23**	13.23	**77.1**	<0.1%	<0.1%	**(K)A D/I G N|A/G V|A|S V S/G S/I/F S R(L)**	854.4379	1707.866	1.5
2	**11.17**	11.17	**70.6**	<0.1%	<0.1%	**(K)F D|L/P V/D S E/H/K(A)**	593.7933	1186.574	4.6

z: peptide charge state. SPI(%): scored peak intensity %, FDR (%): L, local; G, Global. Sequence map: forward-slashes for locations of y-ions; back-slashes for location of b-ions; vertical lines for locations of both b- and y- ions.

Band of ~21 kDa isolated with SDS-PAGE experiment after identification through Western blot analysis performed using the anti-NRT3.1A antibody.

gi|162460401|ref|NP_001105929.1| high affinity nitrate transporter precursor [Zea mays]
MARQQSVHALCVLAALLFAASLPSPAAAGVHLSSLPKALDVTTSAKPGQVLHAGVDSLTVTWSLNATEPAGADAGYKGVKVK**LCYAPAS
QK**DRGWRKSEDDISKDKACQFK**VTEQAYAAAAPGSFQYAVAR**DVPSGSYYLRAFATDASGAEVAYGQTAPTAAFDVAGITGIHASLKIA
AGVFSAFSVVALAFFFVIETRKKNK

Amino acid coverage: 14%.

z	Score	Fwd-Rev Score	SPI (%)	FDR(%) L	FDR(%) G	Sequence Map	Measured m/z (Da)	Matched MH+ (Da)	Error (ppm)
2	**16.64**	16.64	**90.4**	<0.1%	<0.1%	**(K)V T E Q\A|Y|A|A|A|A|P G S F Q Y A V/A R(D)**	1036.02	2071.024	4.1
2	**10.53**	10.53	**66.3**	<0.1%	<0.1%	**(K)L/C|Y/A/P A/S Q/K(D)**	519.2566	1037.508	-2.4

z: peptide charge state. SPI(%): scored peak intensity %, FDR (%): L, local; G, Global. Sequence map: forward-slashes for locations of y-ions; back-slashes for location of b-ions; vertical lines for locations of both b- and y- ions.

Band of ~150 kDa isolated in the nondenaturing Deriphat-PAGE performed using microsomal fraction isolated from maize roots treated with NO_3^- for 8 h.

gi|29412129|gb|AAN05088.1| putative high affinity nitrate transporter [Zea mays]
MAAVGAPGSSLHGVTGREPAFAFSTEHEEAASNGGK**FDLPVDSEHK**AKSVRLFSVANPHMRTFHLSWISFFTCFVSTFAAAPLVPIIRDNLN
LTK**ADIGNAGVASVSGSIFSRLTMGAVCDLLGPR**YGCAFLIMLSAPTVFCMSLIDDAAGYITVRFLIGFSLATFVSCQYWMSTMFSSKIIGT
VNGLAAGWGTMGRRRHAAHMPLVYDVIRKCGATPFTAWRLAYFVPGLMHVVMGVLVLTLGQDLPDGNLRSLQKKGNVNKDSFSKVM
WYAVINYRTWIFVLLYGYCMGVELTTDNVIAEYMYDRFDLDLRVAGTIAACFGMANIVARPMGGIMSDMGARYWGMRARLWNIWILQT

AGGAFCLWLGRASTLPVSVVAMVLFSFCAQAACGAIFGVIPFVSRRSLGIISGMTGAGGNFGAGLTQLLFFTSSTYSTGRGLEYMGIMIMAC
TLPVVFVHFPQWGSMFFPPSATADEEGYYASEWNDDEKSKGLHSASLKFAENSRSERGKR**NVIQADAAATPEHV**

Amino acid coverage: 10%.

z	Score	Fwd-Rev Score	SPI (%)	FDR(%) L	FDR(%) G	Sequence Map	Measured m/z (Da)	Matched MH+ (Da)	Error (ppm)
2	**24.08**	20.58	**98.4**	<0.1%	<0.1%	**(R)N V|I|Q|A|D/A/A|A|T|P E/H V(-)**	718.3628	1435.718	0.5
2	**19.21**	13.76	**95.3**	<0.1%	<0.1%	**(K)A D/I/G N|A|G V|A/S V/S/G/S I/F/S R(L)**	854.4362	1707.866	-0.5
2	**17.49**	12.54	**93.6**	<0.1%	<0.1%	**(K)F D|L|P/V/D/S/E/H/K(A)**	593.7918	1186.574	2.1
2	**11.07**	6.53	**61.6**	<0.1%	<0.1%	**(R)L T|M/G/A/V/C/D L/L/G P R(Y)**	701.8558	1402.718	-9.8

>gi|162460401|ref|NP_001105929.1| high affinity nitrate transporter precursor [Zea mays]
MARQQSVHALCVLAALLFAASLPSPAAAGVHLSSLPKALDVTTSAKPGQVLHAGVDSLTVTWSLNATEPAGADAGYKGVKVK**LCYAPAS
QK**DRGWRKSEDDISKDKACQFK**VTEQAYAAAAPGSFQYAVARDVPSGSYYLR**AFATDASGAEVAYGQTAPTAAFDVAGITGIHASLKIA
AGVFSAFSVVALAFFFVIETRKKNK

Amino acid coverage: 19%.

z	Score	Fwd-Rev Score	SPI (%)	FDR(%) L	FDR(%) G	Sequence Map	Measured m/z (Da)	Matched MH+ (Da)	Error (ppm)
2	**19.18**	19.18	**98.2**	<0.1%	<0.1%	**((K)V T E Q A|Y|A|A|A|A|P G/S F Q Y A V/A R(D)**	1036.0172	2071.024	1.3
2	**15.86**	15.86	**82.5**	<0.1%	<0.1%	**(K)L/C|Y|A|P A/S Q/K(D)**	519.2586	1037.508	1.4
2	**14.08**	6.51	**94.3**	0.80%	0.20%	**(R)D V|P/S/G S Y/Y/L/R(A)**	578.7897	1156.563	7.6

z: peptide charge state. SPI(%): scored peak intensity %, FDR (%): L, local; G, Global. Sequence map: forward-slashes for locations of y-ions; back-slashes for location of b-ions; vertical lines for locations of both b- and y- ions.

Band of ~120 kDa isolated in the nondenaturing Deriphat-PAGE performed using microsomal fraction isolated from maize roots treated with NO_3^- for 8 h and identified through Western blot analysis performed after bidimensional native-/SDS-PAGE using the anti-H^+-ATPase antibody.

gi|803378391|ref|NP_001292776.1| plasma membrane ATPase [Zea mays]
MGGLEEIKNEAVDLENIPIEEVFEQLKCTREGLSSSEGQQRLEIFGPNRLEEKKESKVLKFLGFMWNPLSWVMEMAAIMAIALANGGGKPPD
WQDFVGIIVLLVINSTISFIEENNAGNAAAALMANLAPKTKVLRDGRWGEQEAAILVPGDIISIK**LGDIVPADAR**LLEGDALK**VDQSALTGE
SLPVTK**GPGDEVFSGSTCKQGEIEAVVIATGVHTFFGKAAHLVDSTNQVGHFQQVLTAIGNFCICSIGVGILVEIIVMFPIQHRKYRSGIENLL
VLLIGGIPIAMPTVLSVTMAIGSHKLSQQGAITKRMTAIEEMAGMDVLCSDKTGTLTLNKLSVDKNLVEVFCKGVDKDHVLLLAARASRTE
NQDAIDAAMVGMLADPKEARAGIREIHFLPFNPVDKRTALTYIDADGNWHRVSKGAPEQILDLCHCKEDLRRKVHSIIDKYAERGLRSLAV
ARQEVPEKNKESPGGPWQFVGLLPLFDPPRHDSAETIRKALVLGVNVKMITGDQLAIGKETGRRLGMGTNMYPSSALLGQNK**DATLEALP
VDELIEK**ADGFAGVFPEHKYEIVKRLQEKKHIVGMTGDGVNDAPALKKADIGIAVADATDAARSASDIVLTEPGLSVIISAVLTSRCIFQRM
KNYTIYAVSITIRIVLGFMLIALIWQYDFSPFMVLIIAILNDGTIMTISKDRVKPSPLPDSWKLKEIFATGIVLGSYLALMTVIFFWAMHKTDFFS
DKFGVRSIRDSEHEMMSALYLQVSIVSQALIFVTRSRSWSFVERPGLLLVTAFLLAQLVATFLAVYANWGFARIKGIGWGWAGVVWLYSIV
FYFPLDLLKFFIRFVLSGRAWDNLLENKTAFTTKKDYGREEREAQWATAQRTLHGLQPPEASSNTLFNDKSSYRELSEIAEQAKRRAEIARL
RELNTLKGHVESVVKLKGLDIDTIQQNYTV

Amino acid coverage: 4%.

z	Score	Fwd-Rev Score	SPI (%)	FDR(%)	FDR(%)	Sequence Map	Measured m/z (Da)	Matched MH+ (Da)	Error (ppm)

<table>
<tr><th></th><th></th><th></th><th></th><th>L</th><th>G</th><th></th><th></th><th></th><th></th></tr>
<tr><td>2</td><td>15.54</td><td>15.54</td><td>81.4</td><td><0.1%</td><td><0.1%</td><td>(K)V D/Q\S A/L|T/G E/S/L/P V/T/K(G)</td><td>772.9147</td><td>1544.817</td><td>3.6</td></tr>
<tr><td>2</td><td>11.42</td><td>11.42</td><td>82.7</td><td><0.1%</td><td><0.1%</td><td>(K)L G\D|I|V|P A D/A R(L)</td><td>513.7831</td><td>1026.558</td><td>1.1</td></tr>
<tr><td>2</td><td>9.16</td><td>9.16</td><td>66.9</td><td>4.00%</td><td>0.50%</td><td>(K)D A T\L|E/A|L|P V D E L/I E K(A)</td><td>828.4381</td><td>1655.874</td><td>-3.0</td></tr>
</table>

z: peptide charge state. SPI(%): scored peak intensity %, FDR (%): L, local; G, Global. Sequence map: forward-slashes for locations of y-ions; back-slashes for location of b-ions; vertical lines for locations of both b- and y- ions.

Band of ~240 kDa isolated in the nondenaturing Deriphat-PAGE performed using microsomal fraction isolated from maize roots treated with NO_3^- for 8 h and identified through Western blot analysis performed after bidimensional native-/SDS-PAGE using the anti-H^+-ATPase antibody.

Amino acid coverage: 18%.

>gi|803378391|ref|NP_001292776.1| plasma membrane ATPase [Zea mays]
MGGLEEIKNEAVDLENIPIEEVFEQLKCTR**EGLSSSEGQQRLEIFGPNR**LEEKKESKVLKFLGFMWNPLSWVMEMAAIMAIALANGGGKPP
DWQDFVGIIVLLVINSTISFIEENNAGNAAAALMANLAPKTKVLRDGR**WGEQEAAILVPGDIISIKLGDIVPADARLLEGDALKVDQSALT**
GESLPVTKGPGDEVFSGSTCKQGEIEAVVIATGVHTFFGKAAHLVDSTNQVGHFQQVLTAIGNFCICSIGVGILVEIIVMFPIQHRKYRSGIEN
LLVLLIGGIPIAMPTVLSVTMAIGSHK**LSQQGAITK**RMTAIEEMAGMDVLCSDK**TGTLTLNK**LSVDKNLVEVFCKGVDKDHVLLLAARAS
R**TENQDAIDAAMVGMLADPK**EARAGIREIHFLPFNPVDKRTALTYIDADGNWHRVSKGAPEQILDLCHCKEDLRRKVHSIIDKYAERGLR
SLAVARQEVPEKNKESPGGPWQFVGLLPLFDPPRHDSAETIRK**ALVLGVNVKMITGDQLAIGK**ETGRRLGMGTNMYPSSALLGQNK**DAT**
LEALPVDELIEKADGFAGVFPEHKYEIVKRLQEKKHIVGMTGDGVNDAPALKK**ADIGIAVADATDAAR**SASDIVLTEPGLSVIISAVLTSR
CIFQRMKNYTIYAVSITIRIVLGFMLIALIWQYDFSPFMVLIIAILNDGTIMTISKDRVKPSPLPDSWKLKEIFATGIVLGSYLALMTVIFFWAM
HKTDFFSDKFGVRSIRDSEHEMMSALYLQVSIVSQALIFVTRSRSWSFVERPGLLLVTAFLLAQLVATFLAVYANWGFARIKGIGWGWAGV
VWLYSIVFYFPLDLLKFFIRFVLSGRAWDNLLENKTAFTTKKDYGREER**EAQWATAQR**TLHGLQPPEASSNTLFNDKSSYRELSEIAEQAK
RRAEIARLRELNTLKGHVESVVKLKGLDIDTIQQNYTV

<table>
<tr><th>z</th><th>Score</th><th>Fwd-Rev Score</th><th>SPI (%)</th><th>FDR(%) L</th><th>FDR(%) G</th><th>Sequence Map</th><th>Measured m/z (Da)</th><th>Matched MH+ (Da)</th><th>Error (ppm)</th></tr>
<tr><td>2</td><td>21.88</td><td>21.88</td><td>97.8</td><td><0.1%</td><td><0.1%</td><td>(K)V D/Q|S|A|L|T|G E/S/L|P V/T/K(G)</td><td>772.9097</td><td>1544.817</td><td>-2.9</td></tr>
<tr><td>2</td><td>21.17</td><td>11.99</td><td>90.8</td><td><0.1%</td><td><0.1%</td><td>(K)A D/I/G|I|A|V|A D/A/T/D/A A R(S)</td><td>715.3679</td><td>1429.728</td><td>0.3</td></tr>
<tr><td>2</td><td>17.89</td><td>3.39</td><td>89</td><td><0.1%</td><td><0.1%</td><td>(R)L/S|Q/Q/G A/I/T/K(R)</td><td>473.2754</td><td>945.536</td><td>7.6</td></tr>
<tr><td>2</td><td>17.85</td><td>17.85</td><td>95.7</td><td><0.1%</td><td><0.1%</td><td>(K)L/G|D|I|V|P A D/A/R(L)</td><td>513.7867</td><td>1026.558</td><td>8.1</td></tr>
<tr><td>2</td><td>17.04</td><td>13.22</td><td>94</td><td><0.1%</td><td><0.1%</td><td>(K)D A|T\L|E|A|L|P V D E L I E/K(A)</td><td>828.4417</td><td>1655.874</td><td>1.4</td></tr>
<tr><td>2</td><td>15.78</td><td>15.78</td><td>50.4</td><td><0.1%</td><td><0.1%</td><td>(R)T E N Q\D/A/I/D A A/M/V/G M/L A/D/P/K(E)</td><td>995.4584</td><td>1989.926</td><td>-8.1</td></tr>
<tr><td>2</td><td>14.63</td><td>7.59</td><td>86.9</td><td>3.00%</td><td>0.70%</td><td>(R)L/E|I|F/G/P N/R(L)</td><td>473.2655</td><td>945.515</td><td>9</td></tr>
<tr><td>2</td><td>14.35</td><td>6.3</td><td>75.7</td><td>3.00%</td><td>0.30%</td><td>(K)M/I|T|G D Q L/A/I|G K(E)</td><td>573.8152</td><td>1146.619</td><td>3.8</td></tr>
<tr><td>2</td><td>12.84</td><td>6.84</td><td>89.1</td><td>3.00%</td><td>0.50%</td><td>(K)A L|V|L/G/V/N V/K(M)</td><td>456.8005</td><td>912.588</td><td>6.6</td></tr>
<tr><td>2</td><td>12.06</td><td>12.06</td><td>74.5</td><td><0.1%</td><td><0.1%</td><td>(K)T/G|T|L/T/L/N K(L)</td><td>424.2495</td><td>847.488</td><td>4</td></tr>
<tr><td>2</td><td>11.74</td><td>11.74</td><td>70.8</td><td><0.1%</td><td><0.1%</td><td>(K)A D|G\F|A/G V/F/P E/H K(Y)</td><td>637.816</td><td>1274.616</td><td>6.5</td></tr>
<tr><td>2</td><td>10.45</td><td>10.45</td><td>78.8</td><td>3.00%</td><td>0.30%</td><td>(R)E A Q/W/A/T/A/Q/R(T)</td><td>530.7668</td><td>1060.517</td><td>8.8</td></tr>
<tr><td>2</td><td>10.43</td><td>10.43</td><td>94.6</td><td><0.1%</td><td><0.1%</td><td>(R)E G\L|S/S/S/E/G Q/Q R(L)</td><td>589.2802</td><td>1177.544</td><td>7.4</td></tr>
<tr><td>2</td><td>10.09</td><td>10.09</td><td>75.6</td><td><0.1%</td><td><0.1%</td><td>(R)W G E Q E A/A|I|L|V|P G D I I/S I K(L)</td><td>970.0262</td><td>1939.054</td><td>-4.3</td></tr>
</table>

z: peptide charge state. SPI(%): scored peak intensity %, FDR (%): L, local; G, Global. Sequence map: forward-slashes for locations of y-ions; back-slashes for location of b-ions; vertical lines for locations of both b- and y- ions.

Band of ~700 kDa isolated in the nondenaturing Deriphat-PAGE performed using microsomal fraction isolated from maize roots treated with NO_3^- for 8 h and identified through Western blot analysis performed after bidimensional native-/SDS-PAGE using the anti-H^+-ATPase antibody.

1) >gi|803378391|ref|NP_001292776.1| plasma membrane ATPase [Zea mays]

MGGLEEIKNEAVDLENIPIEEVFEQLKCTREGLSSSEGQQR**LEIFGPNR**LEEKKESKVLKFLGFMWNPLSWVMEMAAIMAIALANGGGKPP
DWQDFVGIIVLLVINSTISFIEENNAGNAAAALMANLAPKTKVLRDGRWGEQEAAILVPGDIISIK**LGDIVPADAR**LLEGDALK**VDQSALTG**
ESLPVTKGPGDEVFSGSTCKQGEIEAVVIATGVHTFFGKAAHLVDSTNQVGHFQQVLTAIGNFCICSIGVGILVEIIVMFPIQHRKYRSGIEN
LLVLLIGGIPIAMPTVLSVTMAIGSHKLSQQGAITKR**MTAIEEMAGMDVLCSDKTGTLTLNK**LSVDKNLVEVFCKGVDKDHVLLLAARAS
R**TENQDAIDAAMVGMLADPK**EARAGIREIHFLPFNPVDKR**TALTYIDADGNWHR**VSK**GAPEQILDLCHCK**EDLRRKVHSIIDKYAERGL
RSLAVARQEVPEKNKESPGGPWQFVGLLPLFDPPRHDSAETIRK**ALVLGVNVKMITGDQLAIGK**ETGRR**LGMGTNMYPSSALLGQNKD**
ATLEALPVDELIEKADGFAGVFPEHKYEIVKRLQEKKHIVGMTGDGVNDAPALKK**ADIGIAVADATDAAR**SASDIVLTEPGLSVIISAVLT
SRCIFQRMKNYTIYAVSITIRIVLGFMLIALIWQYDFSPFMVLIIAILNDGTIMTISKDR**VKPSPLPDSWK**LKEIFATGIVLGSYLALMTVIFFW
AMHKTDFFSDKFGVRSIRDSEHEMMSALYLQVSIVSQALIFVTRSRSWSFVERPGLLLVTAFLLAQLVATFLAVYANWGFARIKGIGWGWA
GVVWLYSIVFYFPLDLLKFFIRFVLSGR**AWDNLLENK**TAFTTKKDYGREER**EAQWATAQR**TLHGLQPPEASSNTLFNDKSSYR**ELSEIAE**
QAKRRAEIARLRELNTLKGHVESVVKLKGLDIDTI**QQNYTV**

Amino acid coverage: 26%.

<table>
<tr><th>z</th><th>Score</th><th>Fwd-Rev Score</th><th>SPI (%)</th><th>FDR(%) L</th><th>FDR(%) G</th><th>Sequence Map</th><th>Measured m/z (Da)</th><th>Matched MH+ (Da)</th><th>Error (ppm)</th></tr>
<tr><td>2</td><td>22.51</td><td>11.28</td><td>96.5</td><td><0.1%</td><td><0.1%</td><td>(K)A D/I|G\I|A|V|A/D/A/T/D A A/R(S)</td><td>715.3706</td><td>1429.728</td><td>4.0</td></tr>
<tr><td>2</td><td>21.25</td><td>21.25</td><td>100</td><td><0.1%</td><td><0.1%</td><td>(K)V D/Q|S|A|L|T|G E|S/L|P V/T K(G)</td><td>772.9141</td><td>1544.817</td><td>2.8</td></tr>
<tr><td>2</td><td>20.20</td><td>20.2</td><td>87.5</td><td><0.1%</td><td><0.1%</td><td>(R)T A|L|T\Y|I|D|A/D/G N/W/H/R(V)</td><td>816.8955</td><td>1632.776</td><td>4.4</td></tr>
<tr><td>2</td><td>19.28</td><td>8.8</td><td>89.1</td><td><0.1%</td><td><0.1%</td><td>(K)M/I|T|G/D/Q L/A/I|G K(E)</td><td>573.8184</td><td>1146.619</td><td>9.4</td></tr>
<tr><td>2</td><td>19.02</td><td>19.02</td><td>88.9</td><td><0.1%</td><td><0.1%</td><td>(K)A D|G|F|A|G/V|F/P E/H K(Y)</td><td>637.8126</td><td>1274.616</td><td>1.2</td></tr>
<tr><td>2</td><td>18.02</td><td>18.02</td><td>88.7</td><td><0.1%</td><td><0.1%</td><td>(R)M T A\I E|E|M|A|G/M/D V/L/C/S D/K(T)</td><td>950.9105</td><td>1900.816</td><td>-1.2</td></tr>
<tr><td>2</td><td>17.83</td><td>17.83</td><td>81.6</td><td><0.1%</td><td><0.1%</td><td>(R)A/W|D/N/L|L/E/N/K(T)</td><td>551.7814</td><td>1102.553</td><td>2.5</td></tr>
<tr><td>2</td><td>17.49</td><td>17.49</td><td>79.3</td><td><0.1%</td><td><0.1%</td><td>(R)T E N Q/D\A|I|D/A/A/M/V|G M/L A/D|P K(E)</td><td>995.468</td><td>1989.926</td><td>1.6</td></tr>
<tr><td>2</td><td>16.57</td><td>13.49</td><td>94.3</td><td><0.1%</td><td><0.1%</td><td>(R)L G/M G T/N|M|Y|P S S A/L/L|G Q/N K(D)</td><td>941.4653</td><td>1881.92</td><td>1.9</td></tr>
<tr><td>2</td><td>16.37</td><td>16.37</td><td>85.3</td><td><0.1%</td><td><0.1%</td><td>(K)G/P/G D|E|V|F/S/G/S/T C K(Q)</td><td>670.7963</td><td>1340.579</td><td>4.9</td></tr>
<tr><td>2</td><td>16.29</td><td>9</td><td>88.6</td><td><0.1%</td><td><0.1%</td><td>(R)E A/Q|W/A/T/A/Q/R(T)</td><td>530.7625</td><td>1060.517</td><td>0.7</td></tr>
<tr><td>2</td><td>15.30</td><td>6.2</td><td>96.3</td><td><0.1%</td><td><0.1%</td><td>(R)L/E|I|F/G/P N/R(L)</td><td>473.2651</td><td>945.515</td><td>8.1</td></tr>
<tr><td>2</td><td>15.04</td><td>15.04</td><td>89.8</td><td>2.00%</td><td>0.90%</td><td>(K)D A\T|L|E|A|L|P V D E L I E K(A)</td><td>828.4374</td><td>1655.874</td><td>-3.8</td></tr>
<tr><td>2</td><td>14.89</td><td>9.57</td><td>90.9</td><td>2.00%</td><td>0.70%</td><td>(K)A L|V|L|G V/N V/K(M)</td><td>456.7985</td><td>912.588</td><td>2.2</td></tr>
<tr><td>2</td><td>14.30</td><td>14.3</td><td>94.3</td><td><0.1%</td><td><0.1%</td><td>(K)L/G|D|I|V|P A D/A R(L)</td><td>513.7822</td><td>1026.558</td><td>-0.7</td></tr>
<tr><td>2</td><td>14.18</td><td>14.18</td><td>76.6</td><td>2.00%</td><td>1.00%</td><td>(R)E L|S/E/I/A/E/Q A/K(R)</td><td>559.2891</td><td>1117.574</td><td>-2.3</td></tr>
<tr><td>2</td><td>14.17</td><td>14.17</td><td>87</td><td><0.1%</td><td><0.1%</td><td>(K)G A/P E\Q/I/L/D/L/C/H/C K(E)</td><td>770.8714</td><td>1540.725</td><td>7.1</td></tr>
<tr><td>2</td><td>13.02</td><td>9.02</td><td>77.5</td><td>2.00%</td><td>0.60%</td><td>(R)V/K|P/S/P L/P D S W K(L)</td><td>627.3514</td><td>1253.689</td><td>5.3</td></tr>
<tr><td>2</td><td>12.63</td><td>12.63</td><td>75</td><td><0.1%</td><td><0.1%</td><td>(K)T/G|T|L/T/L/N K(L)</td><td>424.2502</td><td>847.488</td><td>5.6</td></tr>
<tr><td>2</td><td>12.27</td><td>12.27</td><td>79.6</td><td><0.1%</td><td><0.1%</td><td>(K)G L D\I\D\T\I|Q/Q N/Y/T V(-)</td><td>740.3745</td><td>1479.733</td><td>6.2</td></tr>
</table>

z: peptide charge state. SPI(%): scored peak intensity %, FDR (%): L, local; G, Global. Sequence map: forward-slashes for locations of y-ions; back-slashes for location of b-ions; vertical lines for locations of both b- and y- ions.
