# Supplementary material for: Time-Resolved Investigation of Molecular Components Involved in the Induction of NO3– High Affinity Transport System in Maize Roots
Source: Front Plant Sci. 2016 Nov 8;7:1657. doi: 10.3389/fpls.2016.01657 (PMC5099785; doi:10.3389/fpls.2016.01657)
Supplement: Supplementary file 3 [file Image_1.PDF]

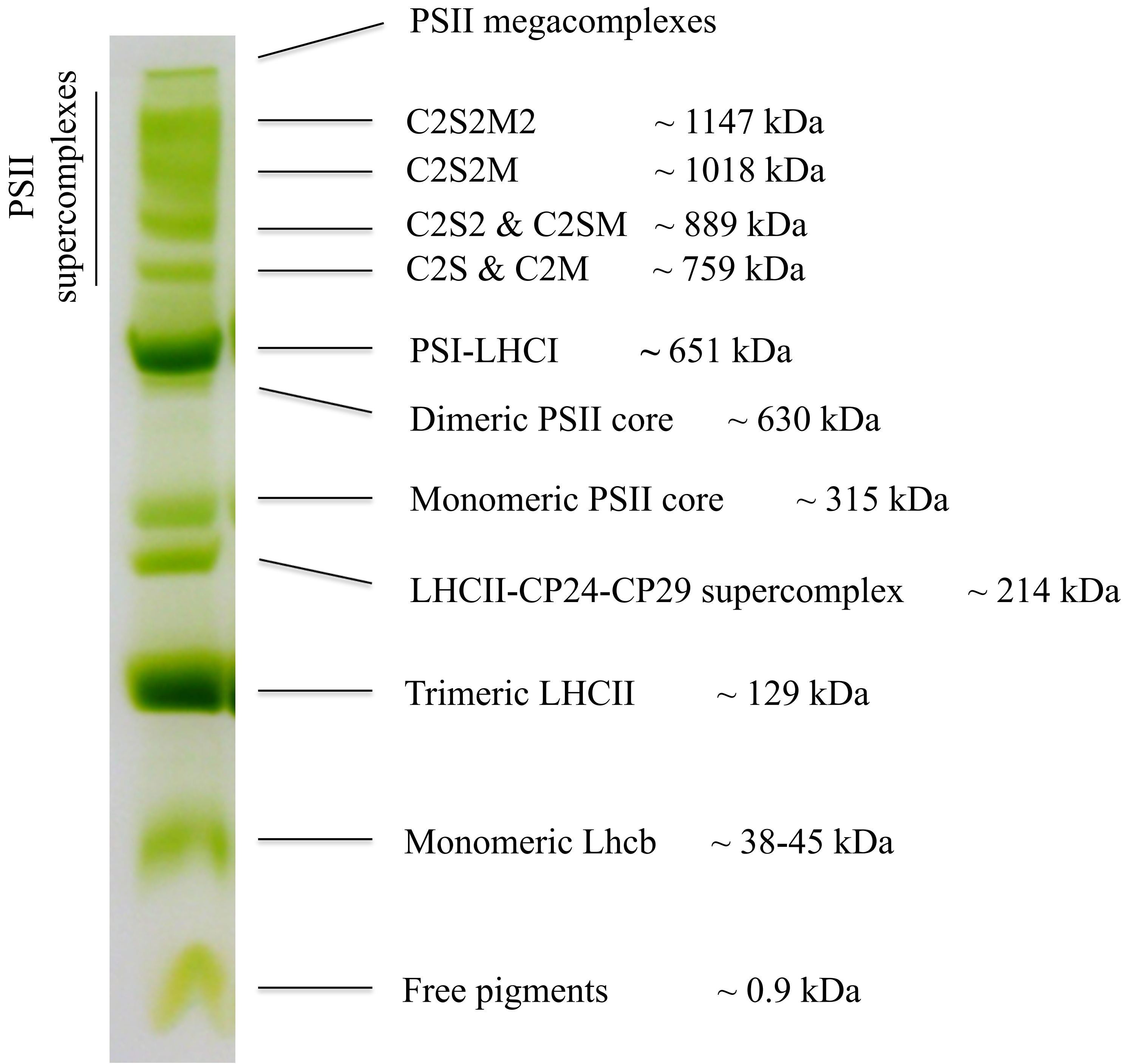
PSII megacomplexes
PSII supercomplexes
C2S2M2 ~ 1147 kDa
C2S2M ~ 1018 kDa
C2S2 & C2SM ~ 889 kDa
C2S & C2M ~ 759 kDa
PSI-LHCI ~ 651 kDa
Dimeric PSII core ~ 630 kDa
Monomeric PSII core ~ 315 kDa
LHCII-CP24-CP29 supercomplex ~ 214 kDa
Trimeric LHCII ~ 129 kDa
Monomeric Lhcb ~ 38-45 kDa
Free pigments ~ 0.9 kDa

Supplementary Figure S1. Fractionation of chlorophyll-binding complexes of the photosynthetic membranes from *Arabidopsis thaliana* leaves. Thylakoid pigment-protein complexes were separated by nondenaturing Deriphat-PAGE upon solubilization with 0.8% α-DM. Fractionation showed 12 green bands, whose nomenclature and composition are indicated on the basis of earlier works (Caffarri et al., 2009; Galka et al., 2012). The molecular weight of complexes were calculated according to the most recent crystal structures solved (Liu et al., 2004; Pan et al., 2011; Umena et al., 2011; Qin et al., 2015).

Caffarri, S., Kouril, R., Kereïche, S., Boekema, E.J., Croce, R. (2009). Functional architecture of higher plant photosystem II supercomplexes. *EMBO J.* 28, 3052–3063. doi:10.1038/emboj.2009.232.

Galka, P., Santabarbara, S., Khuong, T.T., Degand, H., Morsomme, P., Jennings, R.C., Boekema, E.J., Caffarri, S. (2012). Functional Analyses of the Plant Photosystem I–Light-Harvesting Complex II Supercomplex Reveal That Light-Harvesting Complex II Loosely Bound to Photosystem II Is a Very Efficient Antenna for Photosystem I in State II. *Plant Cell* 24, 2963–2978. doi:10.1105/tpc.112.100339.

Liu, Z., Yan, H., Wang, K., Kuang, T., Zhang, J., Gui, L., An, X., Chang, W. (2004). Crystal structure of spinach major lightharvesting complex at 2.72 A resolution. *Nature* 428, 287-292. doi:10.1038/nature02373.

Umena, Y., Kawakami, K., Shen, J.R., Kamiya, N. (2011). Crystal structure of oxygen-evolving photosystem II at a resolution of 1.9A. *Nature* 473, 55-61. doi:10.1038/nature09913.

Pan, X., Li, M., Wan, T., Wang, L., Jia, C., Hou, Z., Zhao, X., Zhang, J., Chang, W. (2011). Structural insights into energy regulation of light-harvesting complex CP29 from spinach. *Nat. Struct. Mol. Biol.* 18, 309-315. doi:10.1038/nsmb.2008.

Qin X, Suga M, Kuang T, Shen JR. (2015). Structural basis for energy transfer pathways in the plant PSI-LHCI supercomplex. Science 348, 989-995. doi: 10.1126/science.aab0214.
